# T249. THE ROYAL AUSTRALIAN AND NEW ZEALAND CLINICAL PRACTICE GUIDELINES FOR SCHIZOPHRENIA AND RELATED DISORDERS (2016) – A STEP TOWARDS BETTER CARE?

**DOI:** 10.1093/schbul/sby016.525

**Published:** 2018-04-01

**Authors:** Cherrie Galletly, David Castle, Frances Dark, Verity Humberstone, Assen Jablensky, Eoin Killackey, Jayashri Kulkarni, Patrick McGorry, Olav Nielssen, Nga Tran

**Affiliations:** 1 The University of Adelaide; 2 University of Melbourne, St Vincent’s Hospital; 3 Metro South Health Services; 4 Northland District Health Board, University of Auckland; 5 The University of Western Australia; 6 Orygen Centre for Youth Mental Health, The University of Melbourne; 7 Monash Alfred Psychiatry Research Centre; 8 St. Vincent’s Hospital

## Abstract

**Background:**

Clinical Practice Guidelines are developed to improve clinical standards, encourage use of evidence-based treatments, and provide a foundation for audits, service evaluation, and research. This presentation by the expert writing group responsible for the updated RANZCP Clinical Practice Guidelines for Schizophrenia and Related Disorders describes the process, the challenges and the barriers in writing these new clinical guidelines. Once published, dissemination, discussion and utilisation of new clinical practice guidelines is crucial.

**Methods:**

The RANZCP Clinical Practice Guidelines (CPG) for Schizophrenia and Related Disorders were developed using the existing RANZCP and international guidelines, research evidence, and in the absence of clear evidence, expert consensus. The NHMRC levels of evidence for intervention studies were used as a benchmark for each recommendation. A clinical staging model was proposed. There was an increased emphasis on physical health comorbidities, psychological treatments, and vocational recovery. The draft document was subjected to extensive review and revision involving independent psychiatrists, other clinicians and stakeholders, consumer groups, RANZCP committees and reviewers for the ANZJP. The Guidelines are available for open access on the RANZCP website at https://www.ranzcp.org/Publications/Guidelines-and-resources-for-practice.aspx.

**Results:**

The Guidelines have been widely cited. The RANZCP has developed a Consumer Guide and Clinical Audit Tools based on the CPG recommendations. The recommendations made in the guidelines have resulted in some controversy – most notably about the use of depot antipsychotics, and antipsychotic medication discontinuation after recovery from first episode psychosis. As with most CPGs, there is no mechanism for ongoing updating of treatment recommendations in response to new evidence, so regular revisions of CPGs will be needed.

**Discussion:**

The Guidelines provide a comprehensive summary of the evidence for interventions to treat schizophrenia and related disorders, set out a recommended standard of care to be adopted by clinicians in Australia and New Zealand, and create a benchmark against which individual practice and services can be compared. The debate generated by the publication of the guidelines has highlighted the gap between the recommended standard of care and existing practice, especially as it relates to the physical care and psycho-social interventions offered to people with these conditions.

